# Supramolecular Tripeptide Hydrogel Assembly with 5-Fluorouracil

**DOI:** 10.3390/gels5010005

**Published:** 2019-01-26

**Authors:** Evelina Parisi, Ana M. Garcia, Domenico Marson, Paola Posocco, Silvia Marchesan

**Affiliations:** 1 Chemical & Pharmaceutical Sciences Department, University of Trieste; Via L. Giorgieri 1, 34127 Trieste, Italy; evelina.parisi@gmail.com (E.P.); anamariagarcia.1988@gmail.com (A.M.G.); 2 Department of Engineering and Architecture, University of Trieste; Via A. Valerio 6/1, 34127 Trieste, Italy; domenico.87@gmail.com (D.M.); paola.posocco@dia.units.it (P.P.)

**Keywords:** peptides, d-amino acids, chirality, hydrogels, drugs, release, self-assembly, 5-fluorouracil

## Abstract

In this work, we present Thioflavin T fluorescence, transmission electron microscopy (TEM), circular dichroism (CD), Fourier-transformed infrared (FT-IR), and oscillatory rheometry studies applied to an antineoplastic drug, 5-fluorouracil (5-FU), embedded in a heterochiral tripeptide hydrogel to obtain a drug delivery supramolecular system. The release of 5-fluorouracil was monitored over time by reverse-phase high-performance liquid chromatography (HPLC) and its interaction with the tripeptide assemblies was probed by all-atom molecular dynamics simulations.

## 1. Introduction

Cancer is one of the most arduous ailments to treat that nowadays affects a large number of people. Although progress has been made in leaps and bounds in research and oncology, the difficulty of treatment persists to this day. The best treatment choice issue is directly correlated to the complexity of the mechanisms involved in the transformation of healthy cells into cancerous ones and their subsequent proliferation and spread to several sites in the body [1]. After a diagnosis of cancer, the treatment of the patient consists of surgery (if applicable in the case of solid tumors) combined with chemotherapeutic drugs and irradiation to reduce the number of malignant cells and tumor reappearance [2,3]. Although skin cancer is not one of the most fatal tumors, it is known to be the most widespread—every year in the United States, the number of patients diagnosed with skin cancer is higher than the number of patients diagnosed with all the other cancers merged together [4]. 

Transdermal drug delivery systems (TDDS) of anticancer drugs are considered appropriate vehicles to allow antineoplastic drugs to perform their action while also limiting common systemic side effects (i.e., fatigue, infection, anemia, nausea and vomiting, diarrhea, etc.) [5]. Different types of nanomaterials have been investigated as TDDS for skin cancer, such as liposomes [6] and niosomes [7], solid lipid nanoparticles (SLNs) [8], polymeric micelles [9] and imprinted nanospheres [10], dendrimers [11], nanotubes [12,13], magnetic nanoparticles [14], and hydrogels [15]. These systems permit the enhancement of the solubility of hydrophobic drugs so as to improve the bioavailability and stability of the antineoplastic compounds and to extend their half-life. In the last decades, the attention to hydrogels as drug delivery systems (DDS) has risen steadily due to their ability to incorporate a high water content and the possibility of a “green” preparation, among other reasons. 

**Figure 1:**
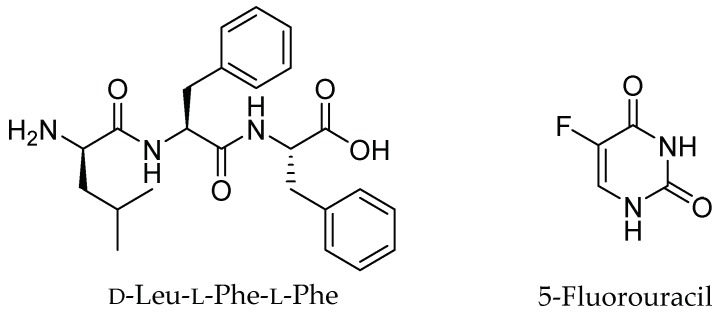
Chemical structures of d-Leu-Phe-Phe and 5-fluorouracil (5-FU).

Supramolecular hydrogels based on short peptides have been studied as convenient vehicles to deliver bioactive molecules and drugs [16] thanks to their facile preparation, low cost, and their biocompatibility and biodegradability, as required for bio-applications [17,18]. The formation of a well-established three-dimensional (3D)-network through a self-assembly mechanism using simple building blocks [19] has been largely exploited to load and release drugs [20]. In this context, the use of a combination of d- and l-amino acids is a useful strategy to increase the resistance against protease activity and to fine-tune self-assembly [21,22,23]. Previous examples of low-molecular-weight gelators formed by heterochiral ultrashort peptides (consisting of just a few amino acids) used as DDS were reported in the literature, including their non-covalent assembly with non-steroidal anti-inflammatory drugs (NSAIDs) for prolonged delivery over time [24,25]. In particular, d-Leu-Phe-Phe (Figure 1) is a well-established hydrogelator that was proven to assemble with the hydrophobic drug ciprofloxacin [26], and, to a minor extent, NSAIDs [25]. The study of supramolecular and biodegradable gel systems is a very important topic in drug delivery, to bring innovation and to provide an alternative to polymers. In addition, despite the fact that various studies have been recently reported in this area, at present there is still a very limited understanding of how such a system should be designed so that a drug can participate in peptide assembly and fibril formation for prolonged release. Isolated examples exist, but clear design rules that identify drug requirements for peptide co-assembly have not yet emerged.

The aim of this work is to further explore the potential of d-Leu-Phe-Phe supramolecular gels for drug delivery, by investigating for the first time its interaction with a hydrophilic drug of a different therapeutic class, and thus with a different chemical structure relative to previous examples. The long-term aim is to gather new insights for the identification of design rules for the co-assembly of drugs and peptides into supramolecular hydrogels. We selected the antineoplastic drug 5-fluorouracil (5-FU) (Figure 1) that acts as an antimetabolite and pyrimidine analog. 5-FU is the backbone of chemotherapy regimens for the medical management of a wide variety of malignancies, including solid tumors for which topical application is relevant. Moreover, the ability of 5-FU to antagonize the immunosuppressive tumor microenvironment makes 5-FU attractive for an additional, positive immunomodulatory effect for treatment consolidation [27]. However, 5-FU suffers from a short biological half-life, and oral formulations have the key drawback of dosage variability, which is particularly important given the low therapeutic index of 5-FU. For this reason, there is ample scope for the development of innovative release formulations based on biodegradable vehicles, especially where local delivery is appropriate [28]. In addition, nanostructured vehicles can have key advantages as they allow the fine-tuning of the experimental partition coefficient of drugs, and they generally display low diffusivity, which is desirable to maintain drugs in the target area when administered locally [29].

5-FU is more hydrophilic than previous drugs tested for assembly with the d-Leu-Phe-Phe peptide hydrogelator. However, aromaticity could be an important factor for enhancing drug interactions with this peptide, and we reasoned that the ability to participate in hydrogen bonding may also favor non-covalent interactions to establish a supramolecular hydrogel system. The fact that the peptide self-assembly also occurs in the presence of large amounts of a hydrophilic component is not to be taken for granted; in a previous study, even small amounts of a hydrophilic component with a diameter of 1–2 nm hindered the assembly of d-Leu-Phe-Phe [30]. Different techniques were used to study the hydrogel, including oscillatory rheology, FT-IR, TEM, and circular dichroism (CD). In this study, we also derived the melting temperature (T_m_) from the CD data to further quantify differences in the gel structure with or without the addition of a drug. Moreover, the drug release process was followed by HPLC and drug–peptide interactions were investigated by molecular dynamics calculations. For the first time, we used in silico studies to visualize the interaction between drug molecules and the surface of a peptide stack, both in parallel and antiparallel arrangements, and they proved useful to confirm the nature, the distance, and the duration of drug–peptide interactions to rationalize experimental observations.

## 2. Results and Discussion

The heterochiral tripeptide d-Leu-Phe-Phe was synthesized and characterized by NMR and LC-MS (Figures S1–S5). It is a well-established gelator at neutral pH in phosphate buffer and has showed promising biocompatibility in vitro [26]. Firstly, the peptide was dissolved at alkaline pH (with or without the drug), thanks to the repulsion between the negative charges in its anionic state. 5-FU stability under the alkaline conditions required for hydrogel preparation was confirmed by NMR analysis (Figures S6–S8). Self-assembly occurred upon the addition of a mildly acidic buffer solution to lower the pH to neutral, the condition at which the peptide is a zwitterion. The final concentration of 5-FU loaded on the peptide-based hydrogel was chosen within the range of topic preparation concentrations already available on the market (0.5% Carac®, 1% Fluoroplex®, 4% Tolak®, 5% Efudex®). 

### 2.1. Hydrogel Characterization

**Figure 2:**
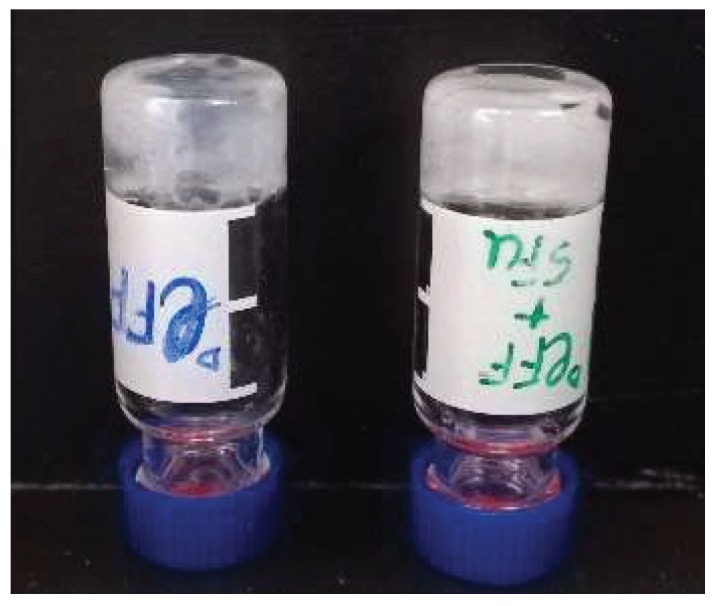
Self-supporting hydrogels obtained: d-Leu-Phe-Phe alone (**left**) and with 5-FU (**right**).

**Figure 3:**
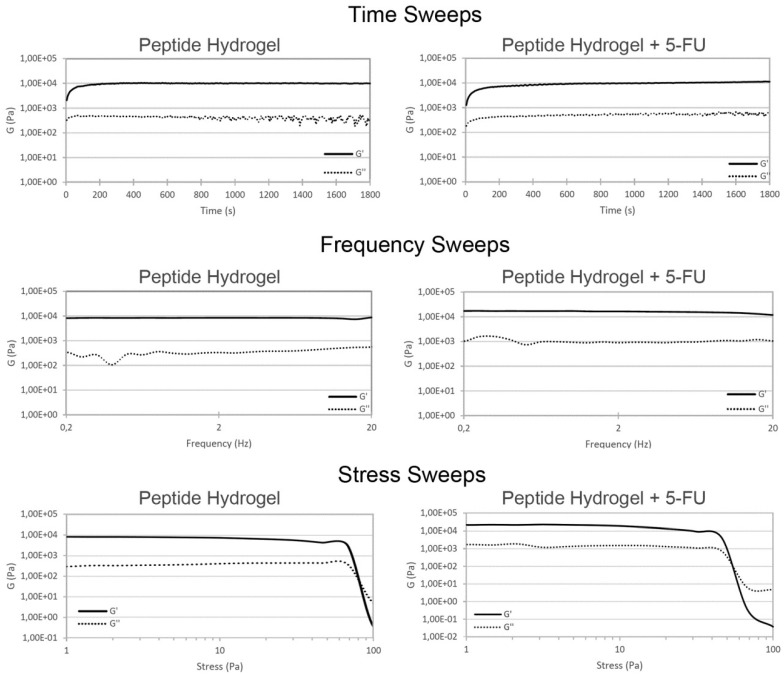
Oscillatory rheometry analysis of hydrogels with the peptide alone (**left**) or with 5-FU (**right**).

The hydrogel obtained from the tripeptide in combination with 5-FU was stable and self-supported (Figure 2) and its viscoelastic nature was assessed by oscillatory rheometry studies (Figure 3). The presence of 5-FU did not give rise to any significant difference in terms of the kinetics of hydrogel formation, which was very rapid in both cases and reached a plateau within approximately 10 minutes. The storage modulus G’ for the peptide hydrogel was slightly increased in the presence of 5-FU (see Figure S9). The stress sweep analysis confirmed a slightly lower resistance for the peptide hydrogel upon drug inclusion, i.e., the crossover that indicates gel-to-sol transition occurs at 55 Pa with the drug and at 80 Pa for the vehicle alone, suggesting a lower level of cross-linking within the gel matrix in the former case (Figure 3, bottom) [31]. 

### 2.2. Nanostructure Morphology

**Figure 4:**
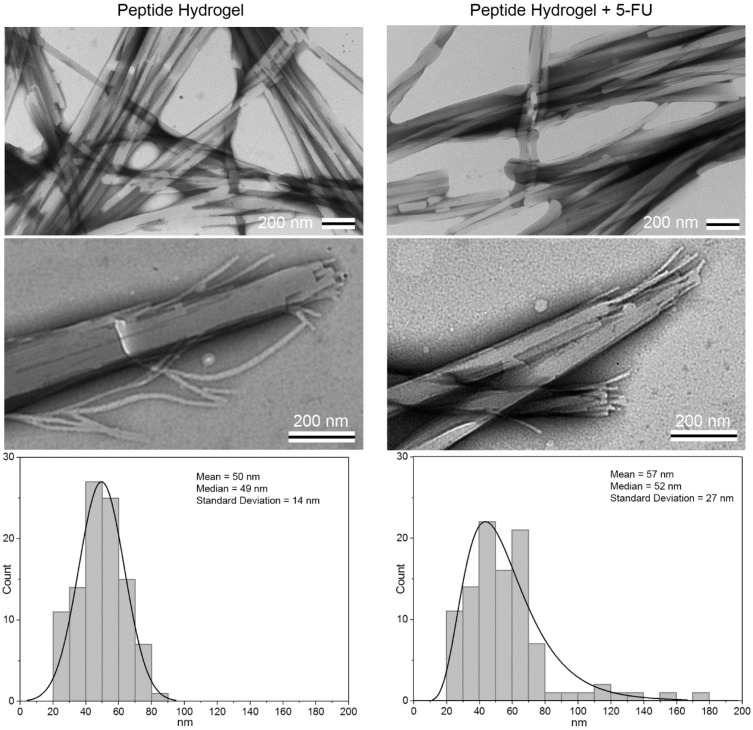
TEM micrographs of peptide hydrogel without (**left**) or with 5-FU (**right**). Median fiber diameters were 49 ± 14 nm and 52 ± 27 nm, respectively (*n* = 100, *p* = 0.02).

TEM was used to study the nanomorphology of each system (i.e., the hydrogel without or with 5-FU) (Figure 4). The overall morphologies of the two materials appeared to be very similar regardless of the presence of 5-FU. Individual fibrils formed by the peptide were 10.3 ± 1.7 nm wide; the inclusion of 5-FU resulted in a very minor but statistically significant (*p* < 0.001) difference in diameter, which was reduced to 9.1 ± 2.1 nm (*n* = 100). In both cases, the hierarchical assembly of fibrils into fibers, either straight or with irregular twists, was evident, as well as their further association into bundles. The median diameter was approximately 50 nm, but samples with 5-FU were more heterogeneous with a wider distribution (Figure 4). Overall, TEM micrographs were comparable to those reported in previous studies for a similar system, whereby the inclusion of NSAIDs resulted in slightly thinner fibrils [25]. 

A convenient method to estimate conformational modifications in a β-rich structure, such as amyloid structures, is the Thioflavin T fluorescence assay (see Figure S10) [32]. The principle behind the test concerns the use of a benzothiazole dye able to bind to the surface of amyloid fibrils, with consequent fluorescence [33]. This phenomenon is due to the limited rotation of the single bond between two aromatic portions of the dye (i.e., the benzothiazole and a substituted benzene) upon non-covalent binding to an amyloid surface [34]. Thioflavin T was able to bind onto the fibers’ surface to yield a noteworthy fluorescence emission, with no significant difference upon the inclusion of the drug (Figure S10). This result indicated that an analogous amyloid surface area was present with or without the drug, consistent with the TEM images that revealed an overall analogous median diameter of the fibers.

### 2.3. Spectroscopic Study of Peptide Conformation

**Figure 5:**
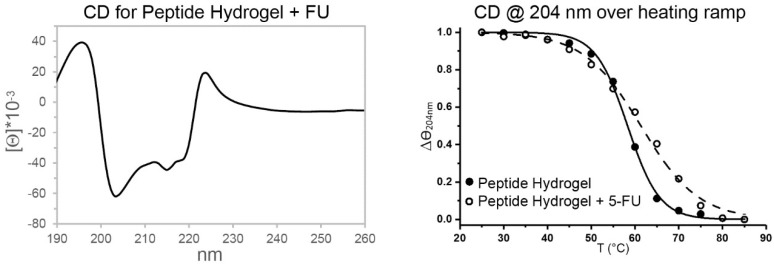
Circular dichroism (CD) spectrum of peptide hydrogel with 5-FU (**left**). CD @ 204 nm over a heating ramp (**right**).

To investigate changes in peptide conformation after the loading of this anticancer drug, circular dichroism (CD) analysis was performed (Figure 5 and Figure S11). The peptide negative signals in the 200–220 nm region due to the amide bond were present with and without the drug, with no significant shifts and only a minor reduction of intensity of the minima at 204 and 215 nm in the presence of 5-FU. CD studies over the first hour of assembly confirmed a very minor effect played by 5-FU, which overall led to a slight decrease in self-assembly kinetics (Figure S11). This is consistent with the rheological and TEM data discussed above, which showed only very minor changes in self-assembly upon drug inclusion.

A heating ramp from 25 to 85 °C was then applied to both systems to reveal the high thermo-resistance of these materials (Figure S12). Surprisingly, the melting temperature (Tm) was slightly increased from 58 to 63 °C upon the inclusion of 5-FU in the system, and overall disassembly occurred over a wider range of temperatures (Figure 5), suggesting a more heterogeneous supramolecular system in the presence of the drug. This finding was in agreement with the TEM observations that revealed a wider distribution of fiber diameters for the drug-loaded hydrogel. 

Fourier-transformed infrared (FT-IR) spectroscopy was also employed to assess peptide conformation (Figure S13). The amide I region did not show significant shifts upon the inclusion of 5-FU in the system, in agreement with the CD and Thioflavin T fluorescence data. We can conclude that peptide conformation was not affected by the presence of 5-FU, although the drug appeared to slightly affect the distribution of fiber diameters, and thus the viscoelastic and thermo-resistance properties. 

### 2.4. Drug Release Studies

**Figure 6:**
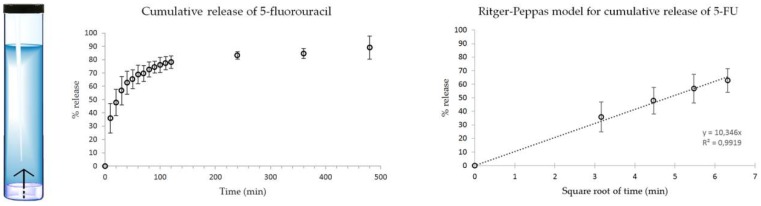
Drug release study for 5-FU monitored by reverse-phase HPLC.

Drug release into phosphate-buffered saline solution at 37 °C was monitored over time, and analyzed by means of high-performance liquid chromatography (HPLC, Figure 6 and Figure S14). 5-FU solubility in water corresponds to 11.1 mg/mL [35], thus the drug was below its solubility limit to maintain sink conditions. The hydrogels were prepared to adhere to the bottom of glass tubes, so that the drug was released along the vertical direction through the surface of the hydrogels into the overlying medium (Figure 6, left). Overall, hydrogel integrity was preserved during the experiment, with no visible swelling. The very fast kinetics of drug release reached a plateau within 8 h, suggesting a limited interaction between the hydrogel matrix and the drug (Figure 6, center). In particular, approximately half of the drug loading was released within the first half-hour, and the majority of the drug was released during the first hour. During this time, the cumulative % drug release followed a linear trend with the square root of time. This release profile was described by the empirical equation of Ritger–Peppas, which can be applied to Fickian one-dimensional diffusion for the initial 60% of cumulative drug release (Figure 6, right). Therefore, the drug was released notably faster relative to other drugs tested previously [25,26], although it should be noted that previous examples concerned drugs with a notably different chemical structure. Nevertheless, drug release for 5-FU followed a kinetics model analogous to that of the naproxen-loaded peptide hydrogel, for which experimental data suggested drug–peptide interactions, although to only a small extent [25]. It would be tempting to simply attribute the limited drug–peptide interaction to drug hydrophilicity, which would prefer to interact with the buffer aqueous solution rather than with the hydrophobic tripeptide. Indeed, 5-FU is notably more hydrophilic (logP −0.89) [36] than ketoprofen (logP 3.12) [37], and naproxen (logP 3.18), which were previously tested for assembly with this peptide into a supramolecular hydrogel for drug delivery [26]. However, ciprofloxacin, which was shown to play a more active role in the assembly of the tripeptide relative to any of the other drugs mentioned above [27], displayed a significantly lower hydrophilicity (logP 0.28) [38]. Therefore, we did not observe any obvious trend between the drug release rate and drug hydrophobicity, suggesting that other factors likely play a role in the drug-peptide interactions. These may include the ability of drugs to engage in specific non-covalent interactions with the peptide. This aspect was thus investigated by means of all-atom molecular dynamics (AA-MD) simulations carried out in explicit water. 

### 2.5. Molecular Modeling of d-Leu-Phe-Phe/5-FU Interaction

Two stacks of six peptides, each in β-sheet organization and arranged side by side, were selected as a minimum representative system for the local d-Leu-Phe-Phe fiber structure [39,40]. Either parallel or antiparallel configurations were considered for the hydrogel arrangement. The ratio between d-Leu-Phe-Phe and 5-FU was chosen to mimic the experimental concentrations, meaning that 16 5-FU molecules were positioned initially close to each d-Leu-Phe-Phe stack. 

**Figure 7:**
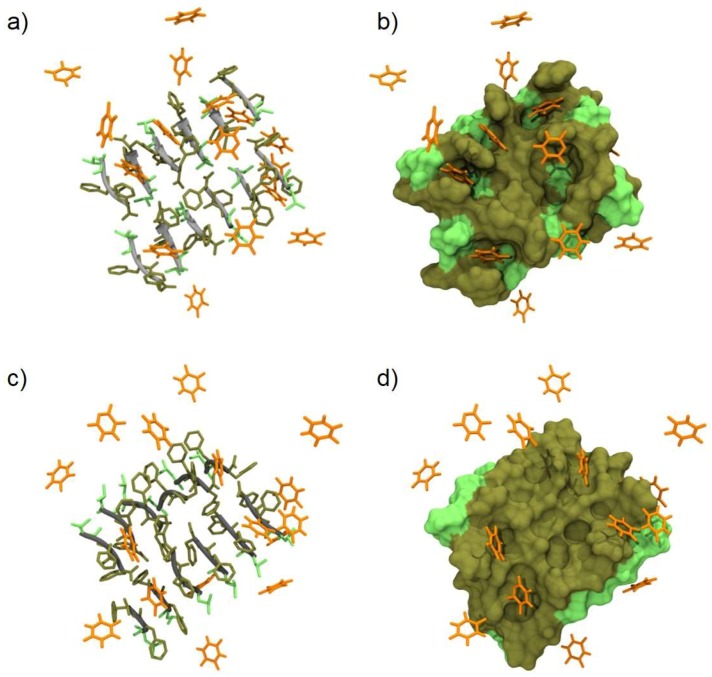
All-atom molecular dynamics (AA-MD) snapshots (**left**) and corresponding molecular surface (**right**) of d-Leu-Phe-Phe stacks in presence of 5-FU. (**a**–**b**) and (**c**–**d**) images refer to antiparallel and parallel β-sheet conformations, respectively. The peptide backbone is depicted in grey and the L/F-side chains are depicted in light and dark green, respectively. 5-FU molecules are represented as orange sticks. Water and ions are not shown for clarity.

Simulations (1 µs long) showed a highly dynamic interaction between the drug and the peptides. Upon inspecting the trajectories, it appeared that the recognition was mainly driven by a π-π stacking between the phenylalanine and 5-FU rings (Figure 7). Transient hydrogen bonds, typically involving the carboxylic acid group and N^1^-H group of the 5-FU, were also detected for each time frame. The weak nature of the π-π stacking and the limited average number of temporary hydrogen bonds can explain the extremely transient interaction and the quick release of the drug from the hydrogel. To gain more insight into this dynamic behavior, we tracked the minimum distance between the heavy atoms (i.e., all not hydrogen atoms) of each 5-FU and d-Leu-Phe-Phe molecule over time (Figure S15). A cut-off range of 5 Å was assigned to define a 5-FU molecule bound to a stack and to count the number of drugs bonded at each time frame (Figure S16). It emerged clearly that 5-FU was able to interact, although weakly, with the peptides either in parallel or antiparallel arrangements. In fact, for most of the 5-FU molecules, the time evolution of the minimum distance fell below the contacting limit of 5 Å but with a highly oscillatory behavior, suggesting a transient and limited affinity of 5-FU toward d-Leu-Phe-Phe. Only for a few 5-FU molecules (as for instance those sampled in Figure S16) the binding event lasted more than 100 ns. On average, six (out of 16) 5-FU molecules stayed in contact with the hydrogel, regardless of its organization, again supporting the weak nature of the interaction forces involved.

## 3. Conclusions

Drug delivery and sustained release over time present several challenges to overcome. This work explored 5-FU, the first example of a hydrophilic drug of very different chemical structure relative to previous cases, as a drug potentially able to interact non-covalently with a self-assembling tripeptide by means of aromatic interactions and hydrogen bonding. These transient interactions were confirmed by in silico studies on peptide stacks, both in parallel and antiparallel arrangements. The overall aim was to assess whether the tripeptide was able to gel in the presence of the drug at levels relevant to those used for therapy, and whether the drug played an active role in assembly for its prolonged release over time. 

Drug-loaded hydrogels were obtained successfully at physiological conditions, with only minor effects on the viscoelastic properties of the final soft materials. TEM micrographs revealed that the overall morphology of peptide assemblies was not altered by the presence of 5-FU, although some minor differences in terms of fibril and fiber size were noted. Circular dichroism, FT-IR, and Thioflavin T fluorescence analyses suggested that peptide conformation was not altered by 5-FU, nor was the resulting amyloid surface. CD supported the rheology and TEM data, as it revealed a minor effect played by the drug on the assembly size distribution, and thus on the hydrogel thermo-resistance.

5-FU release proceeded faster than any other drug tested before with this system [25,26], although no obvious trend between drug release rate and drug hydrophobicity was noted. This observation suggested that other factors come into play. Molecular simulations indeed revealed transient hydrogen bonding as well as π-π interactions, as previously hypothesized for naproxen, which unexpectedly was released following the same kinetic model as 5-FU, although at a slower rate [25]. 

In conclusion, this work showed that antitumoral drugs can also be embedded into a supramolecular hydrogel otherwise composed solely of a tripeptide, and at concentrations that are relevant to those used in the clinic. Despite its hydrophilicity, the aromatic nature of 5-FU as well as its ability to engage in hydrogen bonding led us to believe it may be retained to some extent by the hydrogel matrix. Drug release experiments actually showed that 5-FU was released completely within the first few hours, suggesting the absence of a strong interaction with the peptide assemblies. In silico investigation assisted us in the understanding and quantification of the interactions between drug and peptide molecules that determine the resulting structure, and hence the material behavior. Overall, experimental and theoretical data suggested that π-π interaction and hydrogen bonding were present, although transiently. Future studies will explore other structural features of drug molecules in the quest to identify rules for drug–peptide co-assembly into a supramolecular hydrogel capable of a slow drug release over a number of days.

## 4. Materials and Methods

### 4.1. Materials

The 2-chlorotrytil resin *O*-benzotriazole-*N*,*N*,*N*,*N*-tetramethyl-uronium-hexafluoro-phosphate (HBTU) and Fmoc-protected d-leucine were bought from GL Biochem (Shanghai) Ltd. (Shanghai, China), while Fmoc-protected phenylalanine was bought from Iris Biotech GmbH (Marktredwitz, Germany). All solvents were bought from Merck (Milan, Italy) and were of HPLC grade. Chemical reagents were provided by Sigma (Milan, Italy). Milli-Q-water (MQ water) of high purity and with a resistivity higher than 18 MΩ cm was dispensed by an in-line Millipore RiOs/Origin apparatus (Merck, Milan, Italy). The NMR spectra were recorded on a Varian Innova Instrument (Agilent Technologies, Milan, Italy) at 400 MHz for ^1^H, and at 100 MHz for ^13^C. Tetramethylsilane was used as an internal standard and chemical shifts were reported as ppm. Electrospray MS spectra were obtained with an Infinity 6120 single quadrupole LC-MS system (Agilent Technologies, Milan, Italy). 

### 4.2. Peptide Synthesis and Purification

Peptide synthesis was performed according to standard Fmoc solid phase peptide synthesis (SPPS) with HBTU activation procedures. In short, SPPS was performed in a glass reactor with a sintered glass filter, and Fmoc-amino acid deprotection was achieved with 20% piperidine in *N*,*N*-dimethylformamide (DMF) for 20 min (2 × 10 min) with continuous stirring, until both bromophenol blue and acetaldehyde/chloranil tests turned positive after extensive washings with DMF/DCM. Then, 2.0 equiv. of Fmoc-amino acid was activated in DMF with 1.5 equiv. of HBTU and 1.5 equiv. of HOAt in DMF (4 mL for every equiv. of resin), with the addition of *N*,*N*-diisopropyl ethyl amine (DIPEA) in which 2 mL of a 1 M solution in DMF was added for each equiv. of resin. In the same glass reactor, peptide coupling was attained for 1.5 h at room temperature, and completeness was monitored by colorimetric tests as noted above. The peptide was cleaved from the resin with a mixture of TFA/DCM/TIPS/water (47.5:47.5:2.5:2.5) (TFA: trifluoroacetic acid; TIPS: triisopropyl silane). The resulting solution was concentrated to an oil under air flow, and purification was performed by reverse-phase HPLC (Agilent Technologies, Santa Clara, CA, USA) by dissolving the oil in a mixture of acetonitrile/water. The HPLC Agilent 1260 Infinity apparatus was composed of a preparative gradient pump (1311B), an autosampler (G1329B), a C-18 column (Kinetex, 5 microns, 100 Å, 250 mm × 10 mm, Phenomenex, Torrance, CA, USA), and a Photodiode Array detector (G1315C). The mobile phase was composed of acetonitrile (MeCN) and water with 0.05% TFA with the program as follows: 30% MeCN for the first 2 min; 65% MeCN at *t* = 9 min; 95% MeCN at *t* = 11 min; and finally 95% MeCN was maintained until 14 min (retention time *t*_R_ = 8.5 min). The pure peptide was then lyophilized to a white fluffy powder, and it was identified by ^1^H-NMR, ^13^C-NMR, and ESI-MS.

### 4.3. Hydrogel Preparation

The peptide was dissolved in sodium phosphate buffer (0.1 M pH 12.0), and then the same volume of sodium phosphate at an acidic pH (0.1 M pH 5.8) was added to reach a final peptide concentration of 10 mM (final pH of 7.2 ± 0.1). All buffer solutions were filtered (0.2 µm) prior to use. In the case of the hydrogels containing 5-FU, the peptide was dissolved in sodium phosphate buffer (0.1 M pH 11.8), in which a specific amount (5% of the final volume of the hydrogel) of 5-fluorouracil in 1 M NaOH (from a stock freshly prepared) was added. As last step, half of the final volume of the hydrogel of sodium phosphate monobasic dihydrate (0.1 M pH 4.5) was added to obtain a final pH of 7.3 ± 0.1. The final concentrations of the peptide d-Leu-Phe-Phe and of 5-FU were respectively 10 mM and 13 mM. All buffer solutions were filtered (0.2 µm) prior to use.

### 4.4. Oscillatory Rheometry

A Malvern Kinexus Ultra Plus Rheometer (Alfatest, Milan, Italy) equipped with a 20-mm stainless steel parallel plate geometry was used at the temperature of 25 °C in a Peltier system (Alfatest, Milan, Italy). Hydrogels were assembled in situ and measurements were taken immediately with a gap set to 1.00 mm. Kinetics were studied over 30 minutes at a set frequency of 1.00 Hz and a set stress of 2 Pa. Next, frequency sweeps were obtained with a set stress of 2 Pa, and finally stress sweeps were measured with a controlled frequency of 1 Hz. 

### 4.5. Transmission Electron Microscopy

TEM images were acquired on a Talos F200X, FEI, operating at 80 kV (Thermo Fisher Scientific, Waltham, MA, USA) using TEM grids (copper-grid-supported carbon film). Gels prepared 24 h before were carefully deposited on the grid for TEM analysis. They were then dried at room temperature for 15 min and aqueous tungsten phosphate solution (pH 7.4) was used as a contrast agent. The dimensions of the nanostructures were determined by considering 100 measurements.

### 4.6. Circular Dichroism

A Jasco J-815 Spectropolarimeter (Jasco Europe, Cremella, Italy) was used with one accumulation, 1-s integrations, a bandwidth of 1 nm, and a step size of 1 nm, over a range of wavelengths from 190 to 280 nm at 25 °C. Hydrogel samples were prepared directly in a 0.1-mm quartz cell.

### 4.7. Fourier-Transformed Infrared Spectroscopy

FT-IR spectra were acquired at 1 cm^−1^ resolution and 128 accumulations in a Perkin Elmer System 2000 (Perkin Elmer, Milan, Italy) with the KBr-pellet method. After 24 h of assembly, hydrogel samples were dried in vacuo. The resulting powder was mixed with KBr to obtain a pellet. 

### 4.8. Thioflavin T Fluorescence Assay

The assay was performed in an Infinite M1000 pro (Tecan, Milan, Italy) system using Greiner 96 U Bottom Black Polystyrene plates (VWR, Milan, Italy), into which 150 µL of hydrogels were prepared directly inside the wells. After 1 h of assembly, 30 µL of a solution of Thioflavin T (29.1 µM in 20 mM glycine-NaOH pH 7.5, which was previously filtered with a 0.2-µm filter) was dispensed into the wells. Fluorescence emission was analyzed after 15 min using 446 nm as the excitation wavelength and 490 nm as the emission wavelength (with a 20 nm bandwidth). Each analysis was done in triplicate and the experiment was performed twice (*n* = 6). Microsoft Excel software was used to calculate the average and standard deviations.

### 4.9. Drug Release Assay

For each drug-containing hydrogel, six different samples were prepared as described above (0.250 mL total volume) in a glass tube and were left to self-assemble overnight. The following morning, 4 mL of phosphate-buffered saline (PBS, 0.1 M) was added on top, filling the vials. The vials were incubated at 37 °C, 60 rpm, and at various timepoints. At each timepoint, 50-microliter samples were taken and added to 0.200 mL of water to be analyzed by reverse-phase HPLC, using a Zorbax SB-C18 Rapid Resolution HT 2.1 × 50 mm column at 40 °C (absorbance recorded at 265 nm). The mobile phase consisted of a mixture of H_2_O and MeCN containing 0.05 % TFA. The method used for the analysis was as follows: 0–2 min, 1% MeCN; 2–12 min, 1%–95% MeCN, 12–15 min, 95% MeCN. Each experiment was repeated six times. The average and standard deviation values were calculated and plotted with Excel.

### 4.10. Molecular Modeling

The AMBER-18 suite of software [41] was used for file preparation, running molecular dynamics (MD) simulations, and post-processing analysis in association with VMD software [42] to visualize trajectories and in-house Python scripts for data analysis. More in detail, the d-Leu-Phe-Phe model was parametrized by the AMBER ff14SB forcefield [43]. A well-established procedure [44], in which partial charges are derived by applying the Restrained Electrostatic Potential (RESP method by the RED server [45] and gaff atom types are assigned with antechamber [46,47], was employed to prepare the 5-FU model. Two six-pair stacks of d-Leu-Phe-Phe in antiparallel and parallel beta-sheet conformations were built in VMD based on previous structural evidences [48] (Figure S10). Both systems were then optimized and studied according to the following strategy: a TIP3P [49] water box was created, extending at least 1.5 nm from solute atoms, with Na^+^ and Cl^−^ ions added to neutralize the system charge. A combination of steepest descent (2500 cycles) and conjugate gradient (2500 cycles) minimization steps running on CPU was performed in a first run restraining solute atoms, and then releasing the solute. We then heated the simulation box to 300 K in 20 ps of NVT ensemble simulation, and proceeded in NPT ensemble to equilibrate the system density for another 20 ps. For these first 40 ps of simulation, a 1-fs time integration step was used, and the computation was run on the CPU. Then we switched to a 2-fs integration step and to the GPU version of the code. The systems were equilibrated for 10 ns in NPT ensemble. Berendsen barostat [50], with a pressure relaxation time of 1.5 ps and a target pressure of 1 bar, was used up to this stage, after which we switched to the Monte Carlo barostat implemented in AMBER for the production runs (100 ns). Throughout all the MD simulations, the temperature was controlled by the Langevin method [51] (damping coefficient of 1 ps^−1^), and electrostatic interactions were computed by means of the particle mesh Ewald (PME algorithm. The desired number 5-FU molecules were placed in proximity and within the optimized stacks. Each system was equilibrated according to the procedure described above and simulated for 1 µs of data collection. 

## Supplementary Materials

The following are available online at http://www.mdpi.com/2310-2861/5/1/5/s1.
