# Supplementary material for: Supramolecular Tripeptide Hydrogel Assembly with 5-Fluorouracil
Source: Gels. 2019 Jan 26;5(1):5. doi: 10.3390/gels5010005 (PMC6473331; doi:10.3390/gels5010005)
Supplement: Supplementary file 1 [file gels-05-00005-s001.pdf]

Supplementary Information

Supramolecular Tripeptide Hydrogel Assembly with 5-Fluorouracil

Evelina Parisi [1], Ana M. Garcia [1], Domenico Marson [2], Paola Posocco [2] and Silvia Marchesan [1,*]

[1] Chem. Pharm. Sc. Dept., University of Trieste; Via L. Giorgieri 1, Trieste 34127, Italy, smarchesan@units.it
[2] Department of Engineering and Architecture, University of Trieste; Via A. Valerio 6/1, 34127 Trieste, Italy

* Correspondence: smarchesan@units.it; Tel.: +39-040-558-3923

Table of contents

Supplementary Information 1

1. ^{D}Leu-Phe-Phe spectroscopic data 1

2. Stability of 5-FU in the presence of NaOH 1 M 3

3. Rheometry 5

4. ThT test for amyloid-like structure detection 6

5. Circular Dichroism 6

6. FT-IR spectroscopy 7

7. Drug Release HPLC traces 7

8. ^{D}Leu-Phe-Phe molecular models 12

1. ^{D}Leu-Phe-Phe spectroscopic data

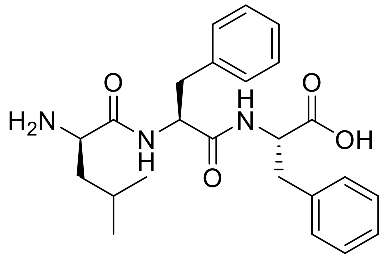

Chemical Formula: $C_{24}H_{31}N_3O_4$
Exact Mass: 425.23
Molecular Weight: 425.53

Figure S1. Chemical structure of ^{D}Leu-Phe-Phe

^{1}H-NMR (400 MHz, DMSO, TMS):δ (ppm) 8.74 (d, *J* = 8 Hz, 1H, NH), 8.60 (d, *J* = 7.8 Hz, 1H, NH), 7.35-7.15 (m, 10H, Ar), 4.71 (m, 1H, αCH), 4.47 (m, 1H, αCH), 3.63 (m, *J* = 6.9 Hz 1H, αCH), 3.10 (m, 2H, βCH), 2.95 (dd, 1H, *J* = 14.0, 9.2 Hz, $βCH_2$), 2.66 (dd, *J* = 13.8; 11.4 Hz, 1H, $βCH_2$), 1.20-1.05 (m, 3H, $βCH_2$ and γ CH), 0.68 (d, 6H, $δCH_3$).
^{13}C-NMR (100 MHz, DMSO, TMS): δ (ppm) 172.7, 171.2, 168.7 (3 x CO); 137.5, 137.4, 129.3, 129.1, 128.3, 128.0, 126.5, 126.3 (10 x Ar); 53.7, 53.6, 50.6, (3 x αC); 40.3 (1 x CH), 38.1, 36.6 (2 x $βCH_2$); 23.1 (γCH) 22.5,

21.7 (2 x δCH_3).

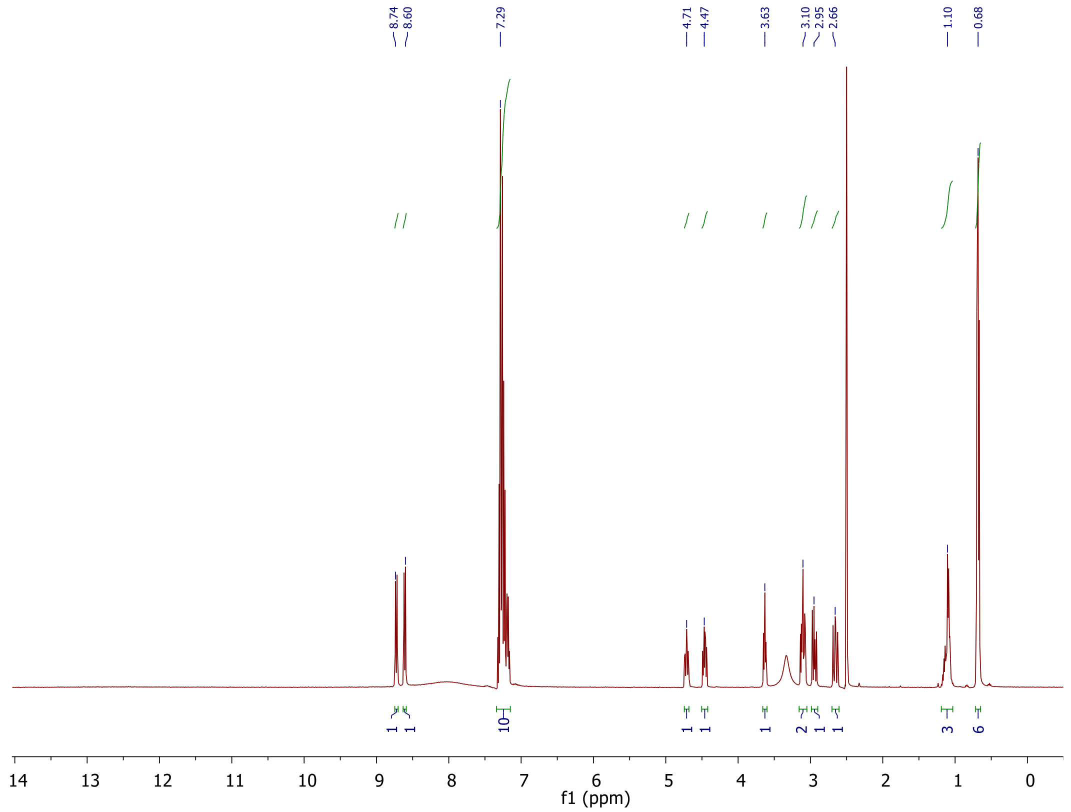

Figure S2. ^{1}H-NMR spectrum of ^{D}Leu-Phe-Phe.

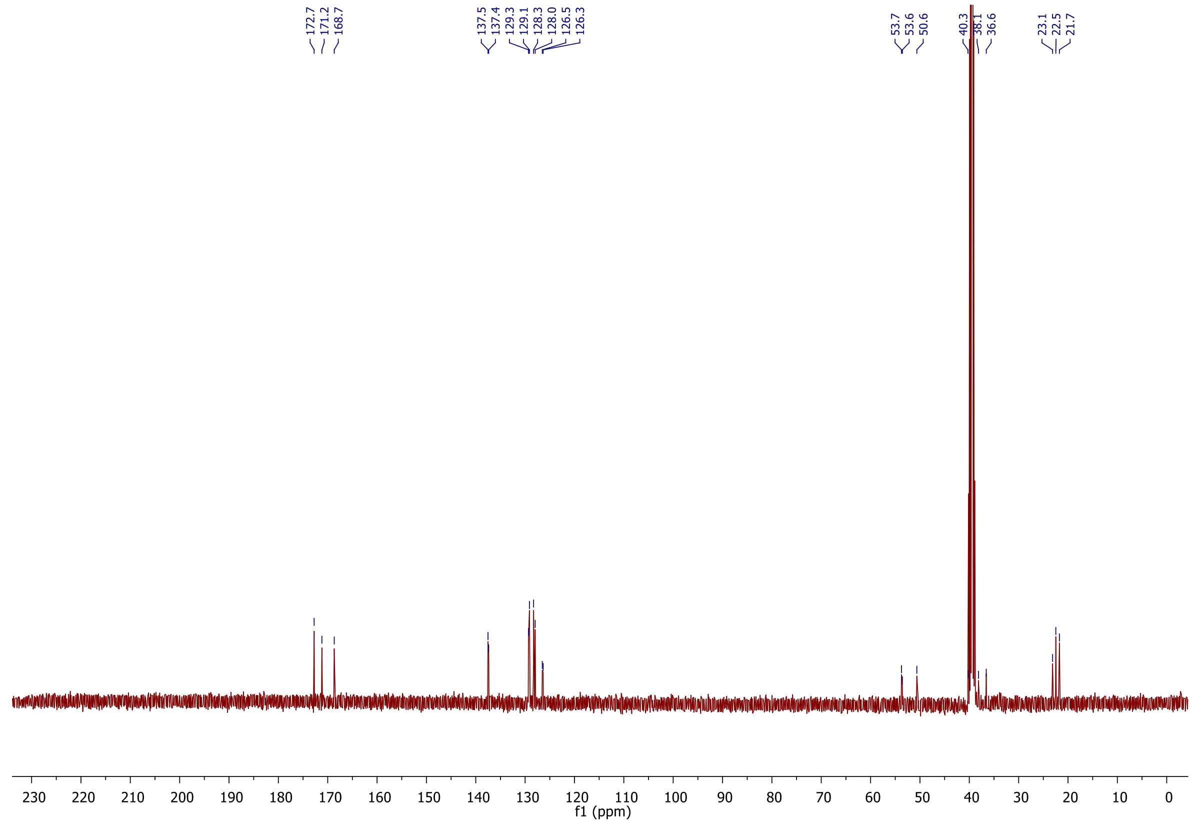

Figure S3. ^{13}C-NMR spectrum of ^{D}Leu-Phe-Phe.

MS (ESI) m/z 426.2 $(M+H)^+$ $C_{24}H_{31}N_3O_4$ requires 426.2.

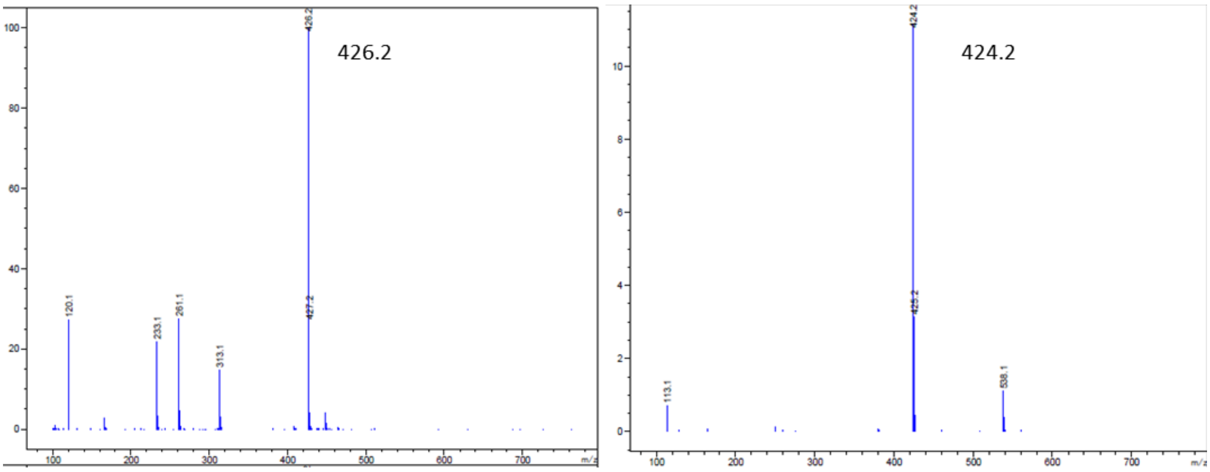

Figure S4. ESI-MS spectra of ^{D}Leu-Phe-Phe in positive (left) and negative (right) ion mode.

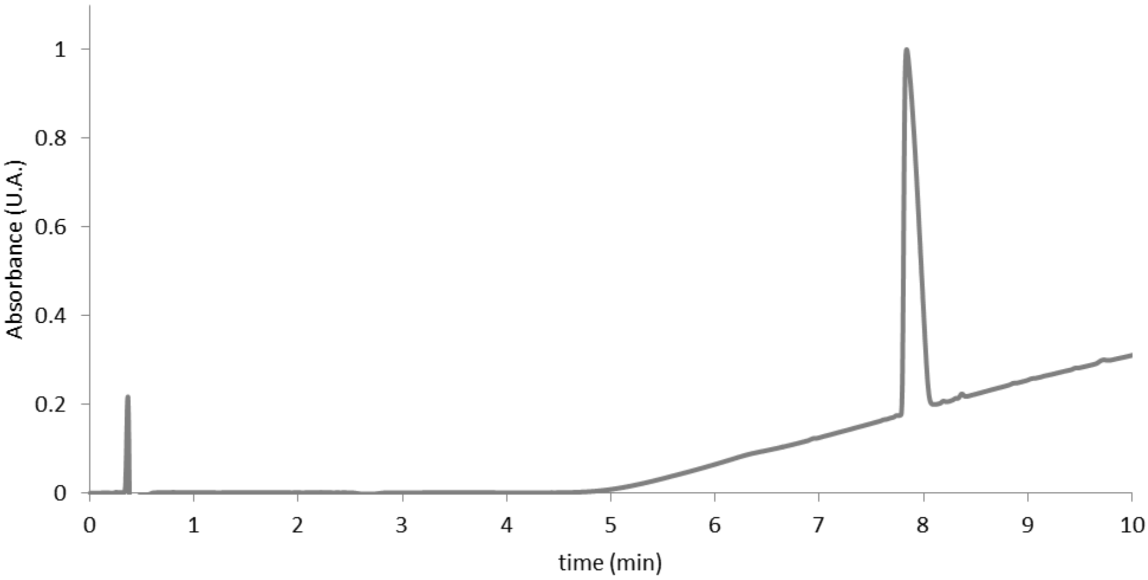

Figure S5. Analytical HPLC trace of ^{D}Leu-Phe-Phe. Column: Zorbax SB-C18 Rapid Resolution HT 2.1x50 mm, particle size: 1.8 microns. Flow 0.5 ml/min. Method: t= 0, 95% water (+0.1% formic acid) and 5% MeCN (+0.1% formic acid); t = 10 min, 5% water (+0.1% formic acid) and 95 % MeCN (+0.1 % formic acid).

2. Stability of 5-FU in the presence of NaOH 1 M

A sample of 5-FU (25 mg) dissolved in NaOH 1M in D_2O (0.75 mL) was analysed by ^{13}C-NMR (12000 accumulations; duration of the experiment: 7 hours). After 24 hours of preparation, ^{1}H- and ^{19}F-NMR analysis was also performed.
^{13}C NMR (100 MHz, D_2O) δ 168.10 (d, *J* = 15.3 Hz, C-2), 165.93 (C-4), 144.20 (d, *J* = 232.8 Hz, C-1), 137.35 (d, *J* = 31.6 Hz, C-6).

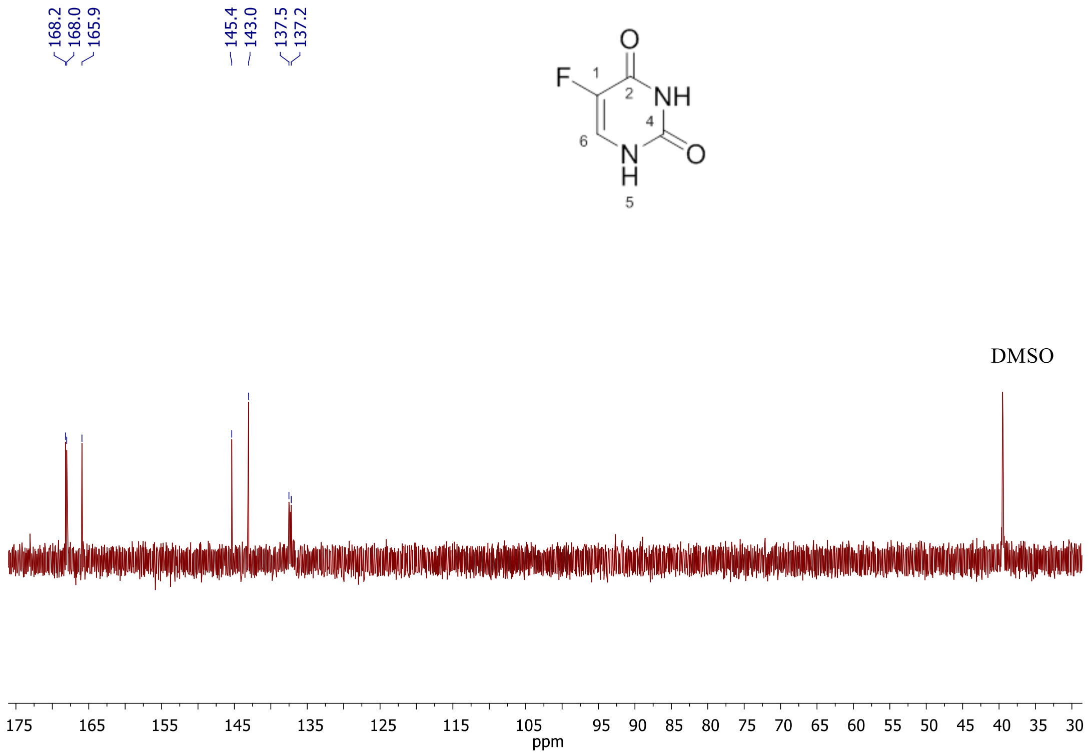

Figure S6. ^{13}C-NMR (12000 accumulations) of 5-FU in NaOH 1M in D_2O (DMSO was used as internal reference).

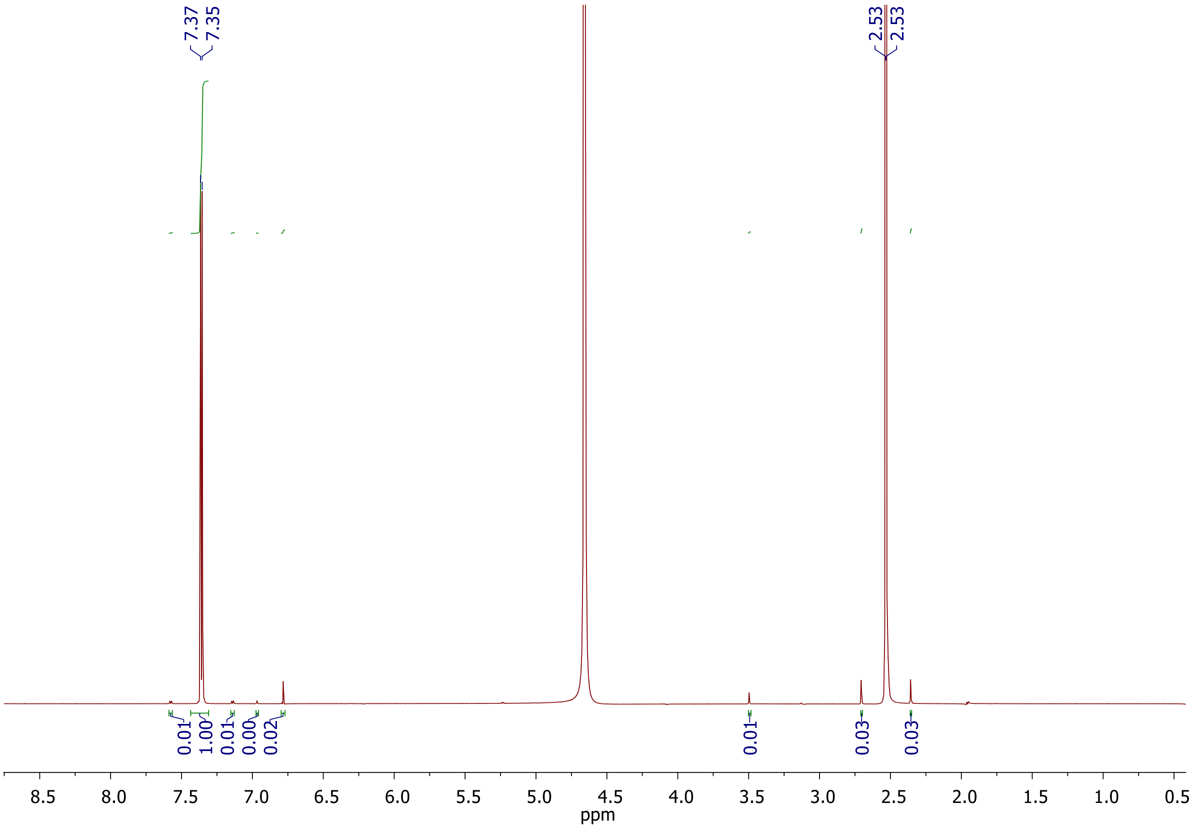

Figure S7. ^{1}H-NMR registered after 24 hours of preparation of 5-FU in NaOH 1M in D_2O. Impurities integrate for less than 3% in relation to 5-FU signal H-6 (δ 7.36 (d, J = 4.7 Hz, 1H)).

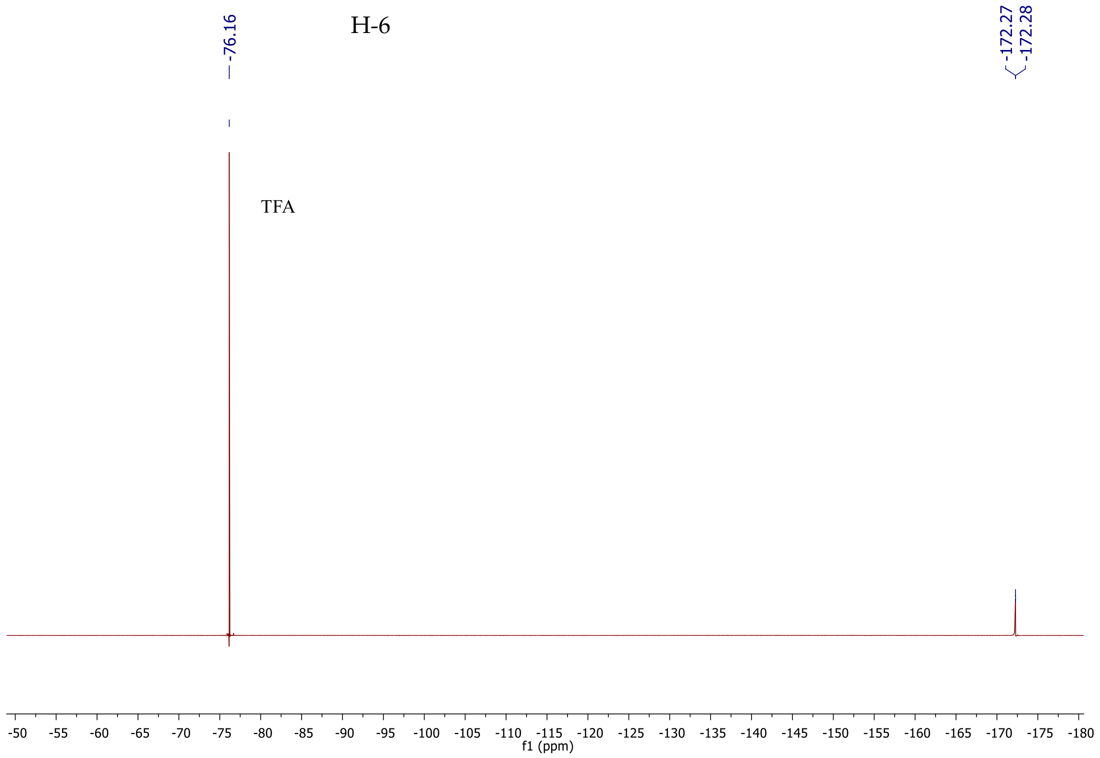

Figure S8. ^{19}F-NMR registered after 24 hours of preparation of 5-FU in NaOH 1M in D_2O (TFA used as internal reference).

3. Rheometry

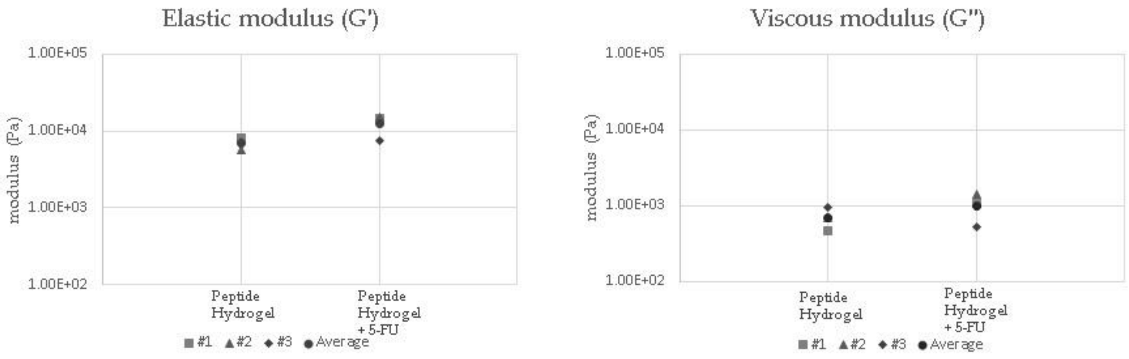

Figure S9. Elastic modulus (G′) and viscous modulus (G′′) of peptide hydrogel without or with 5-FU. Average from three independent measurements.

4. ThT test for amyloid-like structure detection

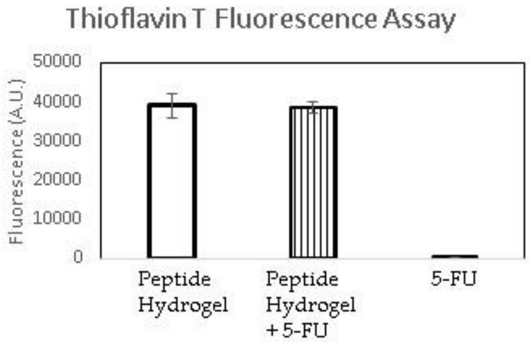

Figure S10. Thioflavin T fluorescence assay.

5. Circular Dichroism

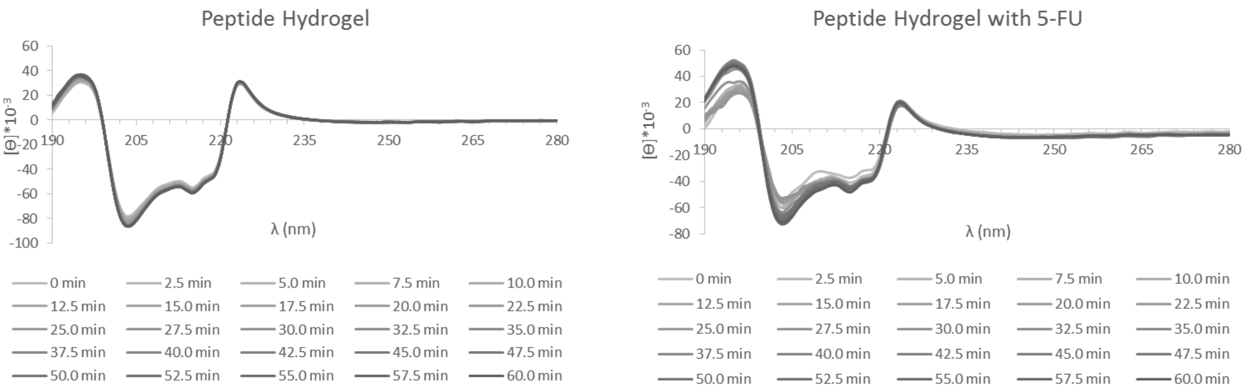

Figure S11. CD spectra of self-assembly kinetics over 60 min of peptide hydrogel (**left**) and peptide hydrogel with 5-FU (**right**).

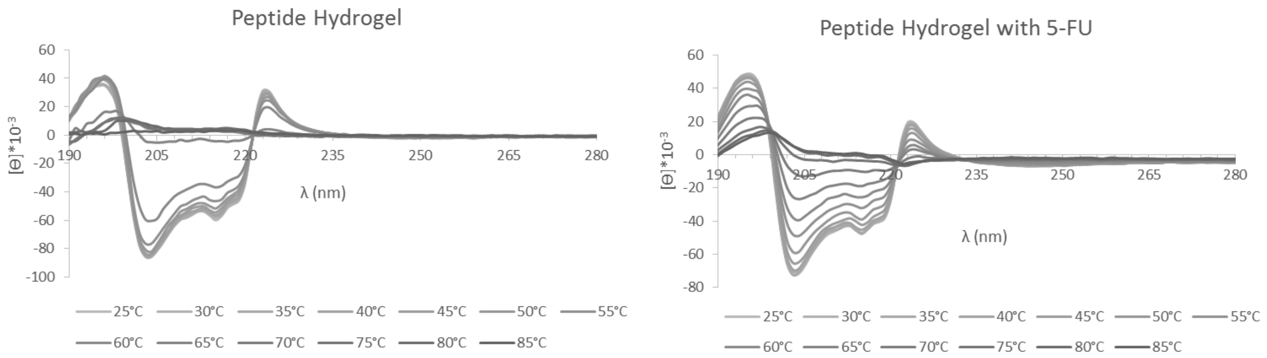

Figure S12. Heating ramp from 25°C to 85°C, after 1h of kinetic for peptide hydrogel (left) and peptide hydrogel with 5-fluorouracil (right).

6. FT-IR spectroscopy

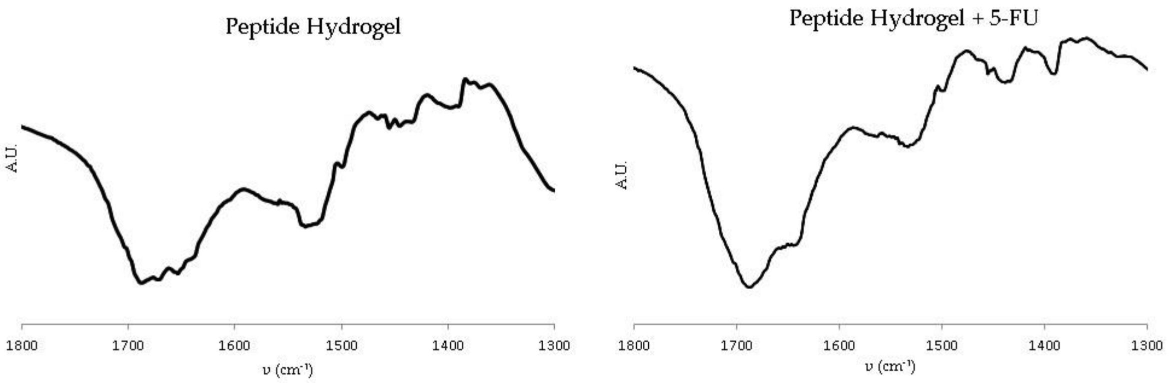

Figure S13. Amide region of FT-IR spectra of peptide hydrogel alone (left) or with 5-FU (right).

7. Drug Release HPLC traces

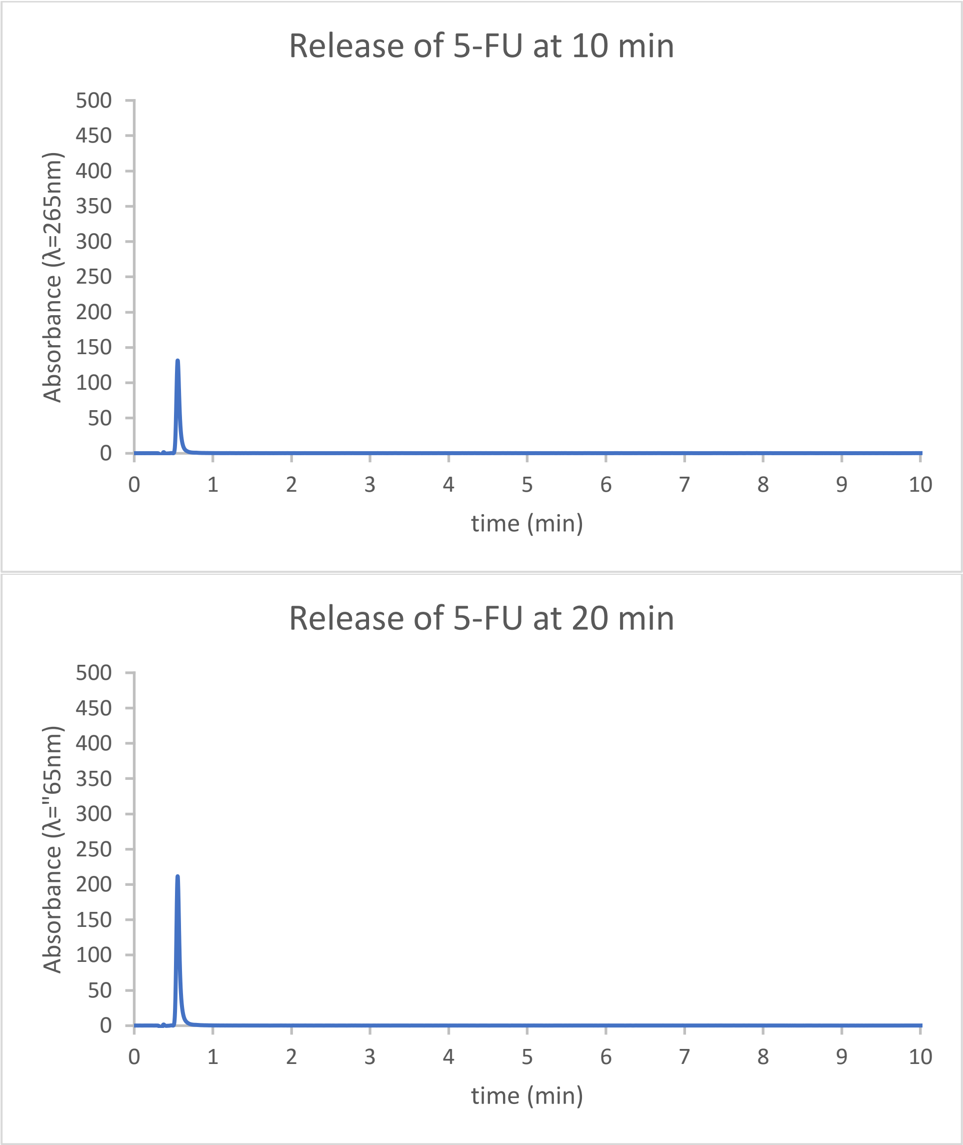

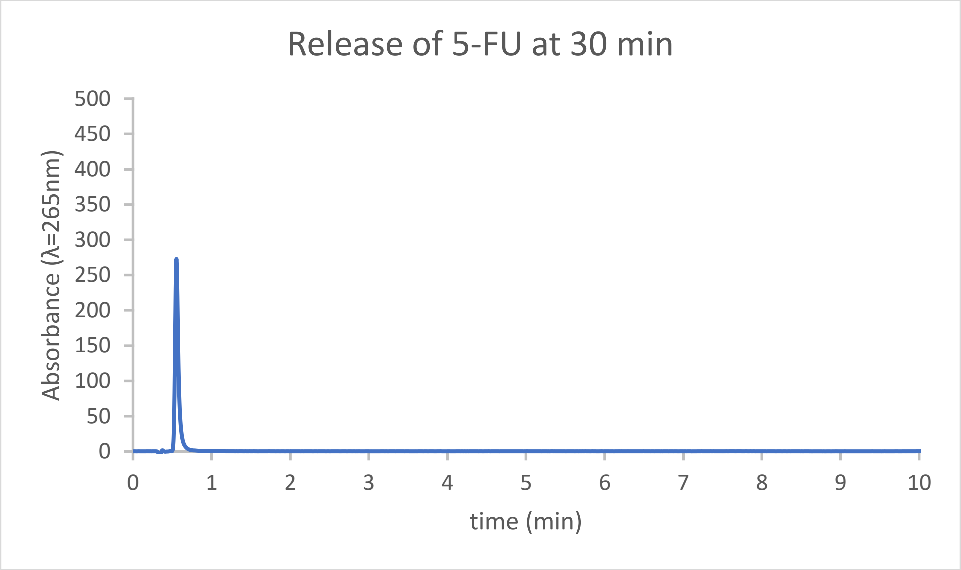
Release of 5-FU at 30 min
Absorbance (λ=265nm)
500
450
400
350
300
250
200
150
100
50
0
0
1
2
3
4
5
6
7
8
9
10
time (min)

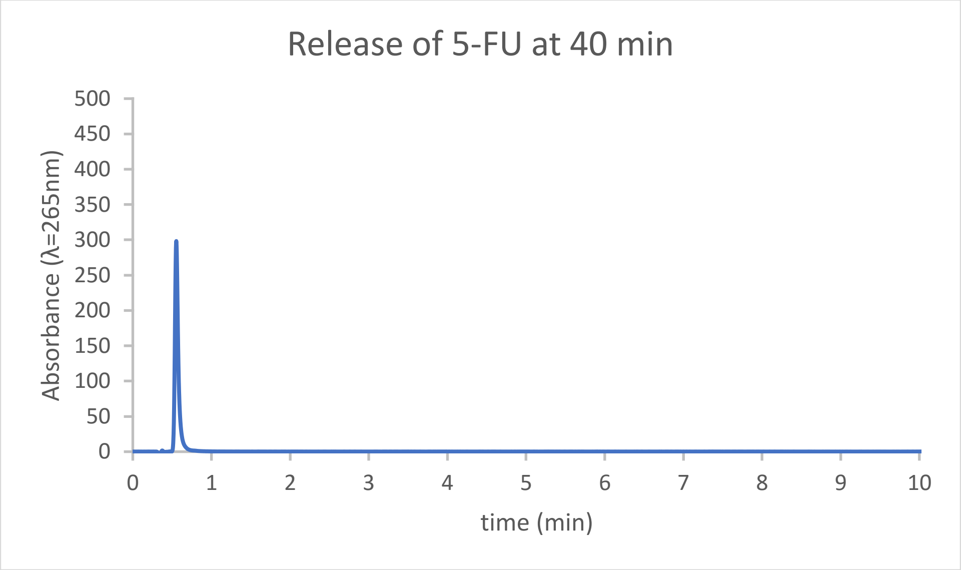
Release of 5-FU at 40 min
Absorbance (λ=265nm)
500
450
400
350
300
250
200
150
100
50
0
0
1
2
3
4
5
6
7
8
9
10
time (min)

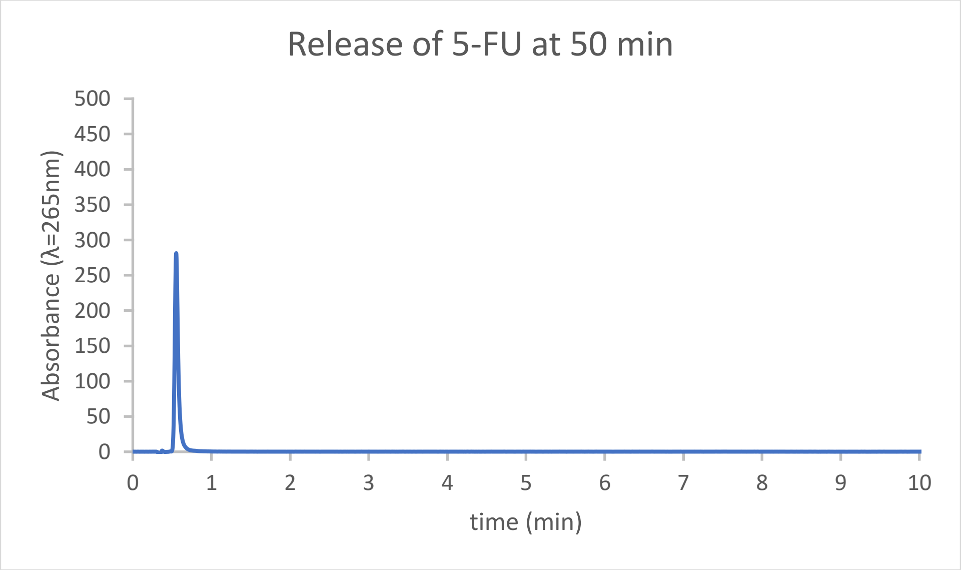
Release of 5-FU at 50 min
Absorbance (λ=265nm)
500
450
400
350
300
250
200
150
100
50
0
0
1
2
3
4
5
6
7
8
9
10
time (min)

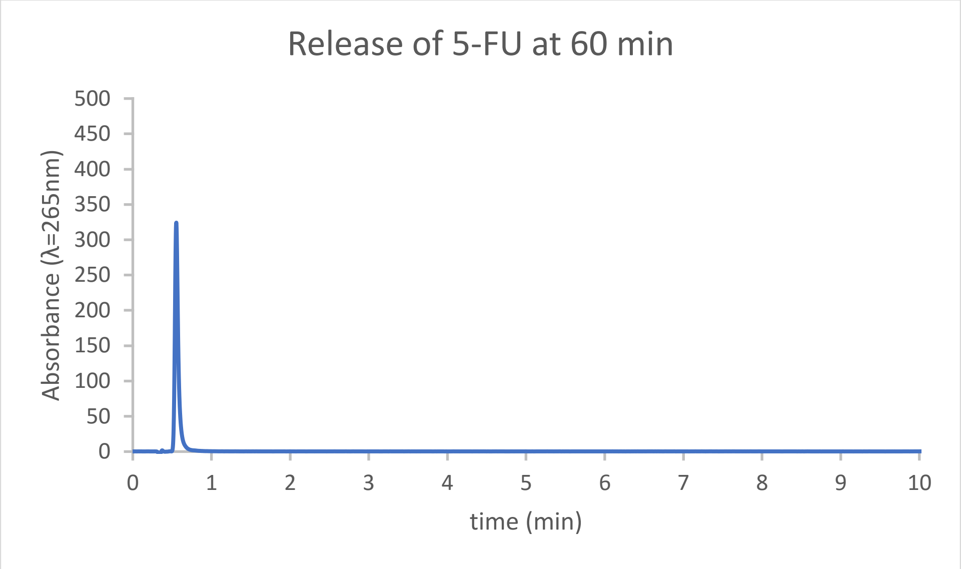
Release of 5-FU at 60 min
Absorbance (λ=265nm)
500
450
400
350
300
250
200
150
100
50
0
0 1 2 3 4 5 6 7 8 9 10
time (min)

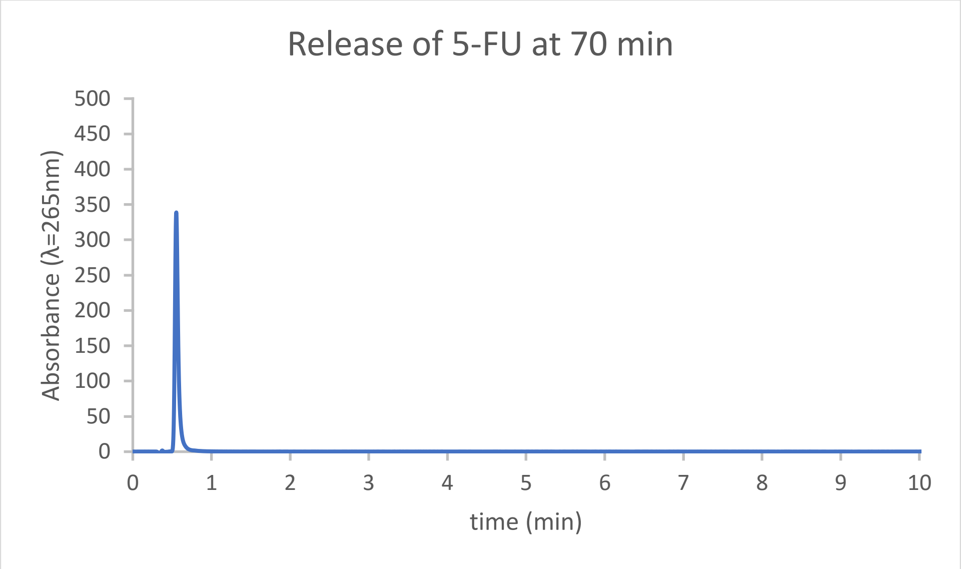
Release of 5-FU at 70 min
Absorbance (λ=265nm)
500
450
400
350
300
250
200
150
100
50
0
0 1 2 3 4 5 6 7 8 9 10
time (min)

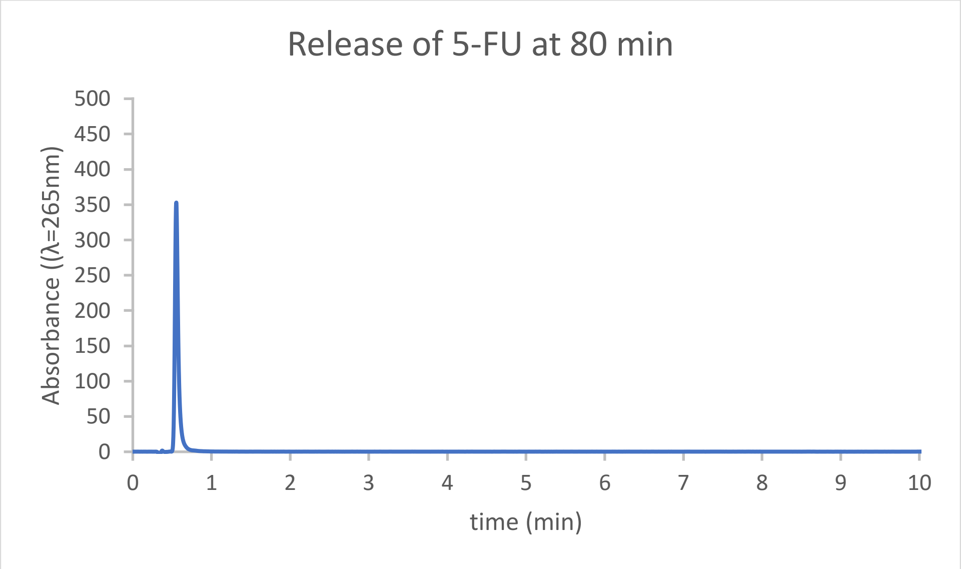
Release of 5-FU at 80 min
Absorbance ((λ=265nm)
500
450
400
350
300
250
200
150
100
50
0
0 1 2 3 4 5 6 7 8 9 10
time (min)

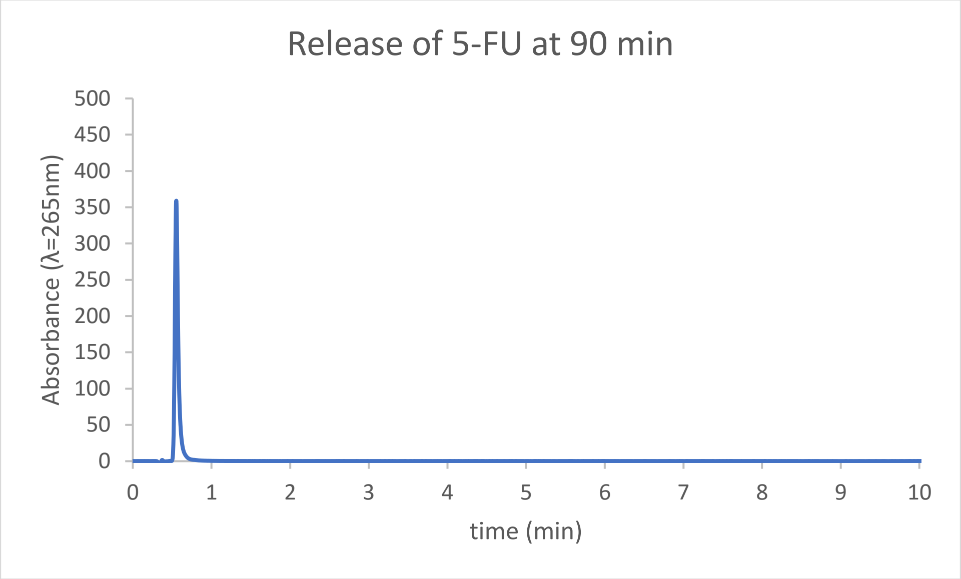
Release of 5-FU at 90 min
Absorbance (λ=265nm)
500
450
400
350
300
250
200
150
100
50
0
0
1
2
3
4
5
6
7
8
9
10
time (min)

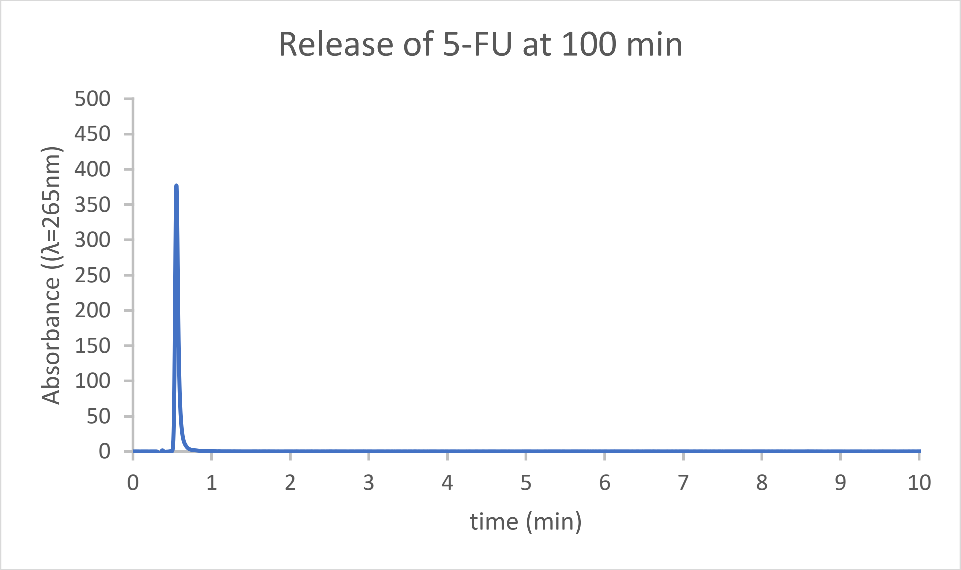
Release of 5-FU at 100 min
Absorbance ((λ=265nm)
500
450
400
350
300
250
200
150
100
50
0
0
1
2
3
4
5
6
7
8
9
10
time (min)

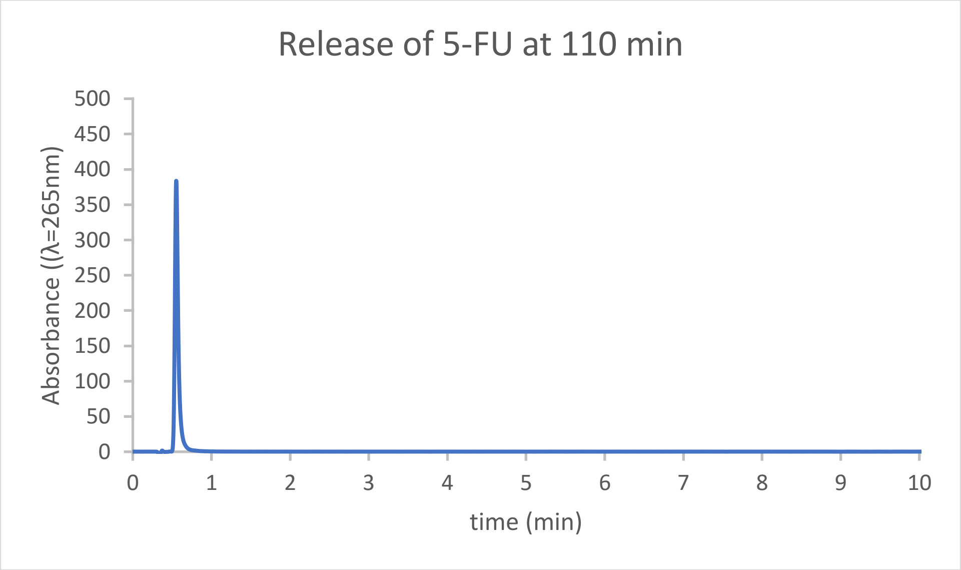
Release of 5-FU at 110 min
Absorbance ((λ=265nm)
500
450
400
350
300
250
200
150
100
50
0
0
1
2
3
4
5
6
7
8
9
10
time (min)

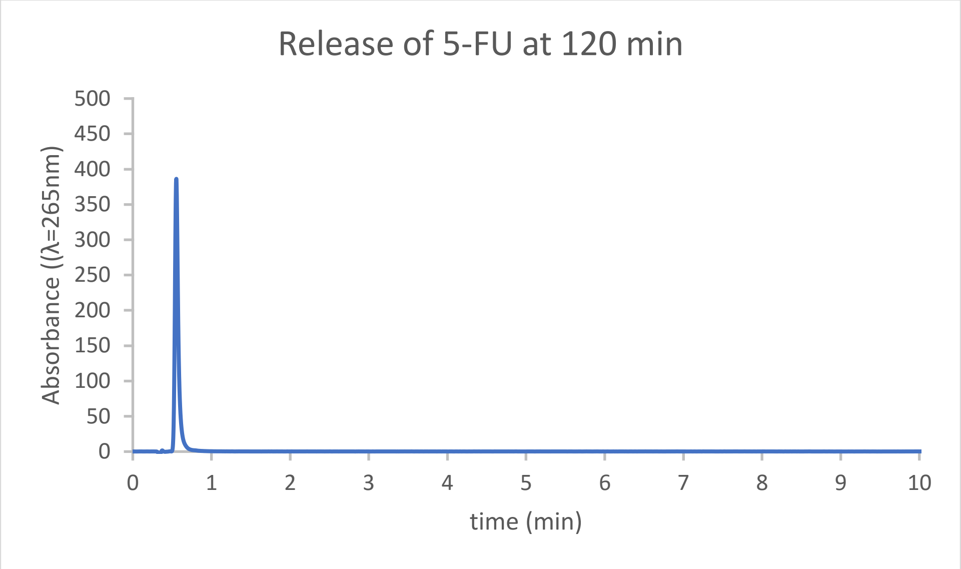
Release of 5-FU at 120 min
Absorbance ((λ=265nm)
time (min)
500
450
400
350
300
250
200
150
100
50
0
0
1
2
3
4
5
6
7
8
9
10

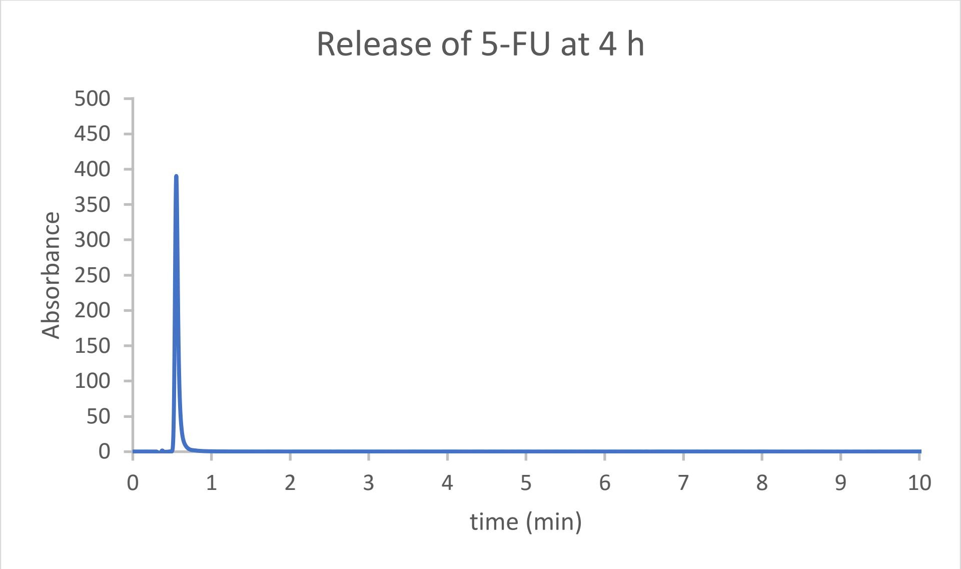
Release of 5-FU at 4 h
Absorbance
time (min)
500
450
400
350
300
250
200
150
100
50
0
0
1
2
3
4
5
6
7
8
9
10

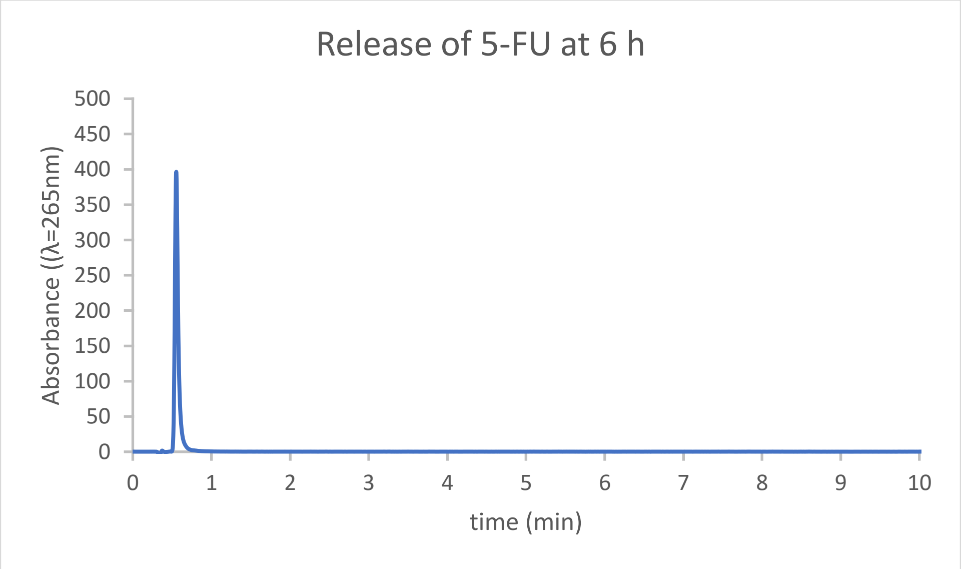
Release of 5-FU at 6 h
Absorbance ((λ=265nm)
time (min)
500
450
400
350
300
250
200
150
100
50
0
0
1
2
3
4
5
6
7
8
9
10

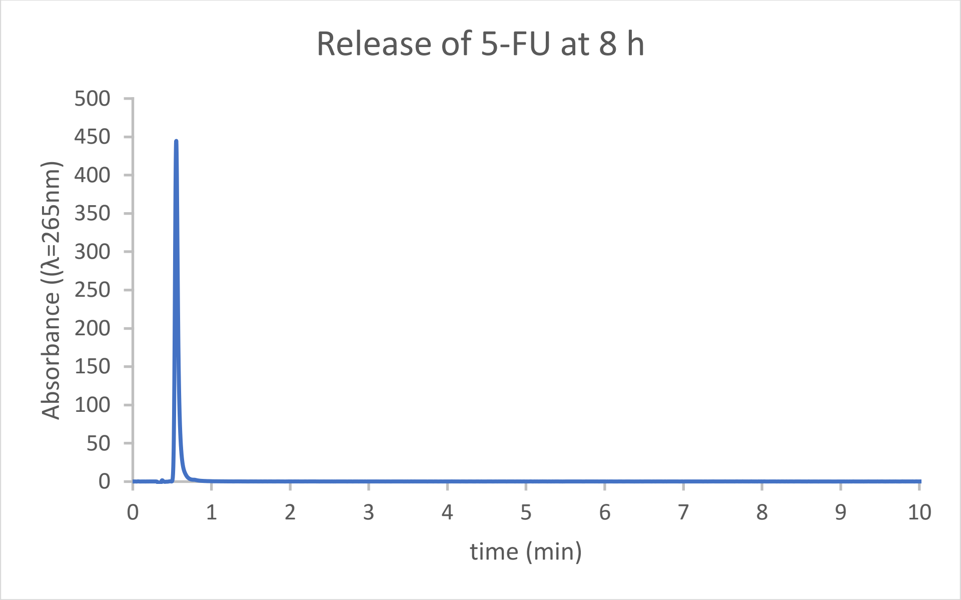

Figure S14. HPLC traces for the drug release over time. *Note:* the injection spike is barely visible relative to the desired signal and occurs at 0.4 min, while 5-FU t_R = 0.6 min.

8. ^{D}Leu-Phe-Phe molecular models

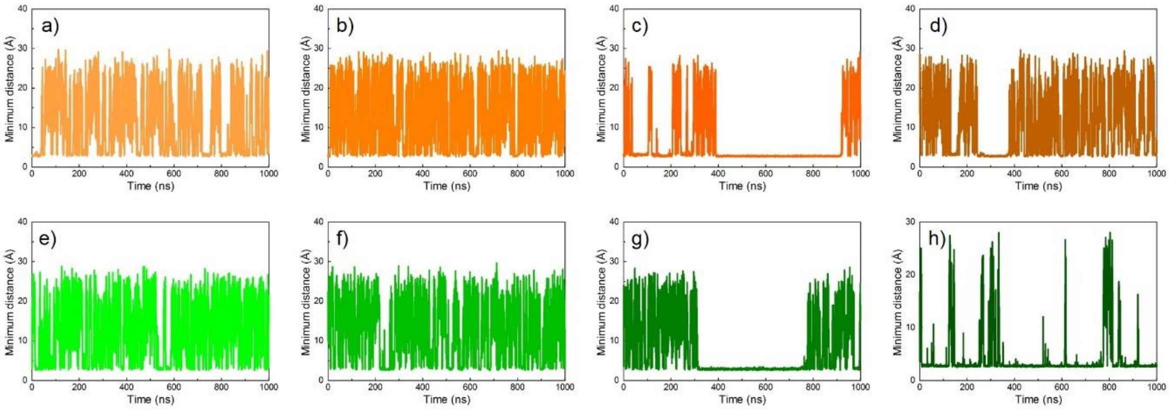

Figure S15. Plot of the minimum distance between selected 5-FU molecules and any heavy atom of the ^{D}Leu-Phe-Phe stack as a function of simulation time. a-d) and e-h) plots refer to antiparallel and parallel β-sheet conformation, respectively.

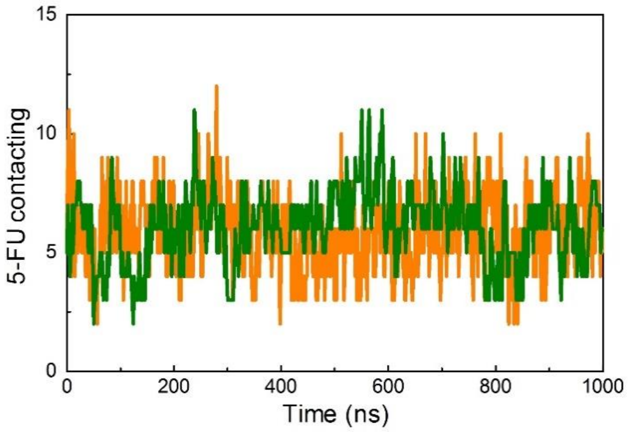

Figure S16. Time evolution of the number of 5-FU molecules contacting anti-parallel (orange) and parallel (green) ^{D}Leu-Phe-Phe stacks. A contact is established when the distance between the drug and the peptide is < 5 Å.
